# A girl with CLOVES syndrome with a recurrent *PIK3CA* somatic mutation and pancreatic steatosis

**DOI:** 10.1038/s41439-019-0063-9

**Published:** 2019-06-24

**Authors:** Hiroaki Hanafusa, Naoya Morisada, Tadashi Nomura, Daisuke Kobayashi, Yoshinobu Akasaka, Ming Juan Ye, Kandai Nozu, Noriyuki Nishimura, Kazumoto Iijima, Hideto Nakao

**Affiliations:** 1 Department of Clinical Genetics, Hyogo Prefectural Children’s Hospital, Kobe, Japan; 2 Department of Pediatrics, Kobe University Graduate School of Medicine, Kobe, Japan; 3 Department of Plastic Surgery, Kobe University Graduate School of Medicine, Kobe, Japan; 4 Department of Orthopaedic Surgery, Hyogo Prefectural Children’s Hospital, Kobe, Japan; 5 Department of Radiology, Hyogo Prefectural Children’s Hospital, Kobe, Japan; 6 Department of Neonatology, Hyogo Prefectural Children’s Hospital, Kobe, Japan

**Keywords:** Pre-diabetes, Disease genetics

## Abstract

CLOVES syndrome is characterized by congenital lipomatous overgrowth, vascular malformation, epidermal nevi, and scoliosis/spinal malformation. It is caused by somatic mosaicism of gain-of-function variants of *PIK3CA*. Here, we describe a novel case of a 5-year-old Japanese girl with CLOVES and concurrent pancreatic steatosis. She had a recurrent somatic mutation in *PIK3CA* (NM_006218.3: c.1357G>A, p.Glu453Lys), elevated HbA1c levels, and pancreatic steatosis. This case indicates that pancreatic screening is critical for *PIK3CA-*related disorders.

CLOVES syndrome (MIM #612918) is a rare disorder, and the abbreviation stands for congenital lipomatous overgrowth, vascular malformation, epidermal nevi, and scoliosis/spinal malformation^1^. CLOVES syndrome is generally caused by somatic mosaicism of gain-of-function variants in *PIK3CA* (3q26.32). In a previous report^2^, mutant allele frequencies were found to vary in the affected tissues, although the mutation rate in peripheral blood was very low. Therefore, it is important to analyze DNA derived from affected tissues and not from the peripheral blood for the diagnosis of CLOVES syndrome.

Pancreatic steatosis (PS), also called pancreatic lipomatosis, is frequently found in the adult pancreas and is typically benign. PS is identified by histological analysis or imaging, e.g., hyperechogenicity on abdominal ultrasonography and hypodensity of the pancreas on computed tomography^3^. PS is associated with obesity, increased age, and diabetes mellitus (DM)^4^. PS rarely occurs during childhood, and some genetic disorders have been associated with the development of PS^4^. However, to the best of our knowledge, PS has not been reported in patients with CLOVES syndrome. In this report, we describe a pediatric patient with CLOVES syndrome with concurrent PS.

The patient was a 5-year-old Japanese girl who was the second child of nonconsanguineous healthy parents. At 26 weeks of gestation, she was diagnosed with left pleural effusion and fetal hydrops by ultrasonography, and pleural drainage was performed. She was delivered by emergency cesarean section at 33 weeks of gestation because of increasing fetal hydrops. Her Apgar score was 5 (1 min)/7 (5 min), and her birth weight was 2604 g (> 97th percentile). She showed scattered capillary malformations in the upper and lower lips and both upper and lower limbs and enlargement of both second toes at birth. At 5 years, she was referred to our outpatient department. At that time, she showed systemic lipomatous overgrowth, particularly in the thorax and legs. Ultrasonography revealed lipomatous overgrowth and no development of mammary gland tissue. Scoliosis was observed by spinal X-ray examination (Fig. 1a). Enlargement of both second toes was still observed. Brain magnetic resonance imaging showed no abnormality, and she had no developmental disorders. Accordingly, we considered a diagnosis of CLOVES syndrome. Abdominal ultrasonography showed pancreatic hyperechogenicity (Fig. 1b), indicating PS. Her hemoglobin A1c (HbA1c) was elevated (6.1%). Her height at 5 years of age was 108.6 cm (− 0.7SD), and her body weight was 21.9 kg (body mass index, 18.6).

**Fig. 1:**
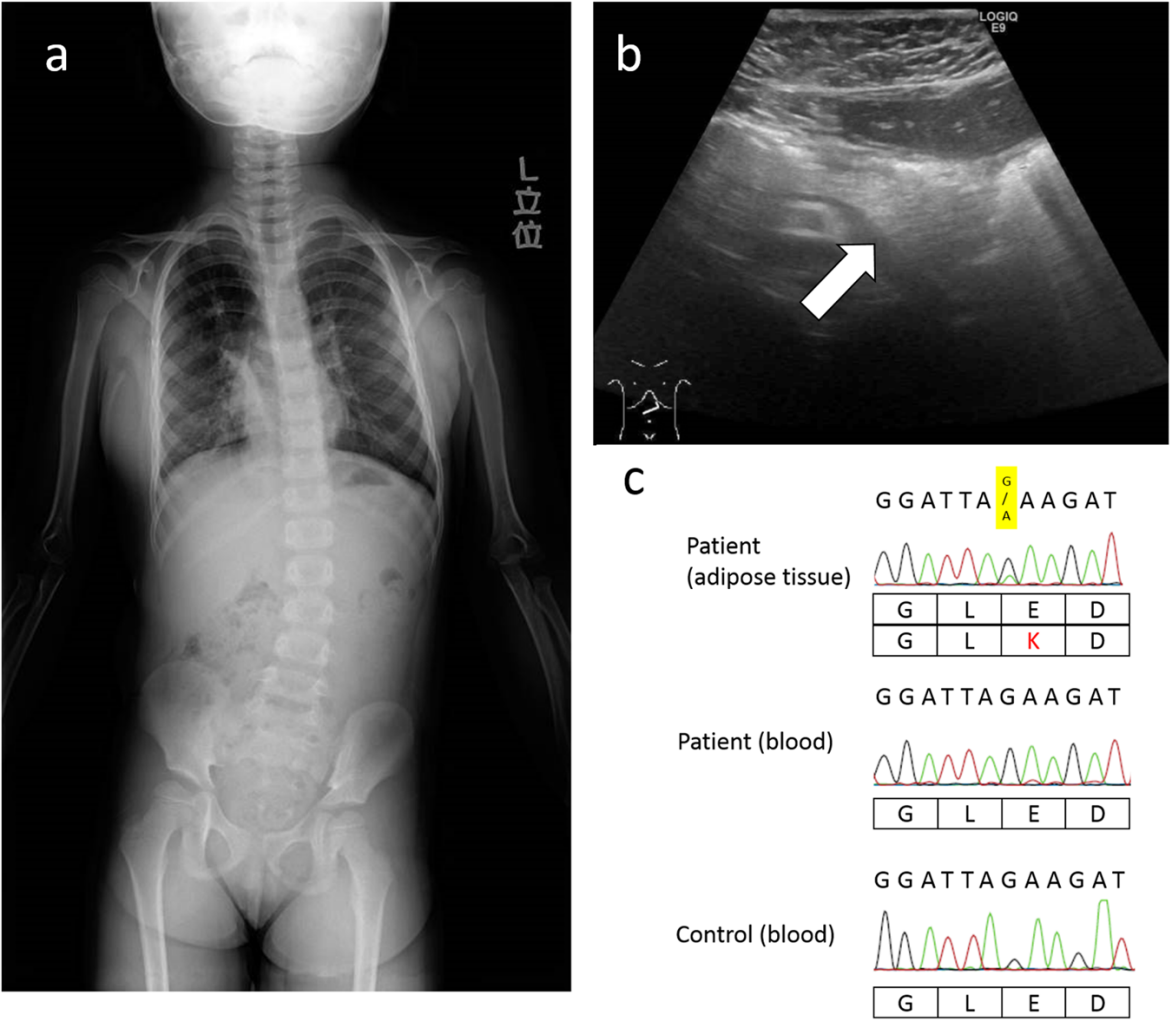
**a** Chest radiograph of the patient showed scoliosis. **b** Ultrasonography showed a hyperechogenic pancreas (white arrow). **c** Results of *PIK3CA* gene analysis by Sanger sequencing. Identification of a mosaic missense variant of *PIK3CA* (c.1357G>A causing p.Glu453Lys) in DNA derived from skin fibroblasts; no variant was observed in DNA derived from peripheral blood

To confirm her molecular diagnosis, we analyzed DNA samples derived from peripheral blood and affected adipose tissues using Sanger sequencing to detect the *PIK3CA* variant after obtaining written informed consent from her parents. All procedures were reviewed and approved by the Institutional Review Board of the Kobe University School of Medicine and were performed in accordance with the ethical standards of the Declaration of Helsinki. We identified a heterozygous missense variant in *PIK3CA* (NM_006218.3: c.1357G>A, p.Glu453Lys) in DNA derived from affected adipose tissue, although the same variant was not found in DNA derived from peripheral blood (Fig. 1c). Mirzaa et al.^5^ reported four patients with c.1357G>A diagnosed with megalencephaly-capillary malformation–polymicrogyria (MCAP) syndrome or somatic overgrowth, including CLOVES syndrome. Finally, we diagnosed the patient with CLOVES syndrome. To consider the mosaic ratio, we performed droplet digital polymerase chain reaction (ddPCR)^6^. We used 100 ng of template DNA from adipose tissue and peripheral blood for ddPCR using a QX200 Droplet Generator, QX200 Droplet Reader, and QuantaSoft software (Bio-Rad, Hercules, CA, USA), and the mutant allele frequencies were found to be 29.7% and 0.27%, respectively.

*PIK3CA* encodes a 110 kDa catalytic subunit of phosphatidylinositol-3-kinase (PI3K). The PI3K/AKT/mammalian target of rapamycin pathway is involved in cell proliferation and cell growth. Somatic *PIK3CA* variants cause abnormal activation of this pathway, and patients with *PIK3CA* aberration show overgrowth syndromes (e.g., CLOVES syndrome and MCAP syndrome). The umbrella term “*PIK3CA*-related overgrowth spectrum (PROS)” has been proposed^7^. Mirzaa et al.^5^ hypothesized that phenotypic differences in overgrowth syndromes caused by somatic mosaic *PIK3CA* variants may depend on the variant site and mosaic frequency. Piacitelli et al.^6^ reported a patient with different mosaic ratios of *PIK3CA* p.Glu545Lys by tissue analysis and found no correlation between tissue-specific disease severity and mutant allele frequencies. We identified the mutant allele frequency of the patient to be 29.7% in affected tissue and 0.27% in white blood cells by ddPCR; hence, it is possible that mosaic frequency or differences in affected tissues might be involved. Further studies are required to explain these phenotypic differences.

The specific etiology of PS remains unclear. *PIK3CA* gain-of-function variants lead to overgrowth of fat tissues. Thus, patients with PROS are at risk of PS. Most patients with PS show a benign clinical course, but some may exhibit DM, pancreatic ductal failure, and pancreatic cancer. Our patient already showed elevated HbA1c levels, indicating that she was at high risk of DM and pancreatic cancer. Therefore, when clinicians encounter patients with PROS, pancreatic abnormalities should also be assessed.

In conclusion, our case demonstrated that pancreatic screening is essential for patients with PROS. Recently, specific therapy for PROS has been reported^8^; thus, it is necessary to establish approaches for the definitive diagnosis of PROS.

## Data Availability

The relevant data from this Data Report are hosted at the Human Genome Variation Database at 10.6084/m9.figshare.hgv.2585.
